# Measurement of NAD(H) concentration using BCA dye and applications to assays of dehydrogenases

**DOI:** 10.17912/micropub.biology.001831

**Published:** 2025-11-19

**Authors:** Umanga Rupakheti, Derian Andrew, Sara E Scanlan, Jackson Tester, Christopher E Berndsen

**Affiliations:** 1 Department of Biology, James Madison University, Harrisonburg, Virginia, United States; 2 Department of Chemistry and Biochemistry, James Madison University, Harrisonburg, Virginia, United States; 3 Biotechnology Program, James Madison University, Harrisonburg, Virginia, United States

## Abstract

Dehydrogenases are a widespread enzyme family that play essential roles in all metabolic processes. We observe that one of the substrates of many dehydrogenases, the coenzyme NAD(H), reacts with copper in the bicinchoninic acid assay to produce a color change that can be measured spectroscopically. NADH reacts across a lower concentration range in this assay (<1 μM to ~50 μM) compared to NAD ^+^ (>100 μM), which allows the bicinchoninic acid (BCA) assay to be used to measure dehydrogenase activity, as demonstrated in assays with human malate dehydrogenase 1.

**Figure 1. Measurement of NADH concentration in a BCA assay:**
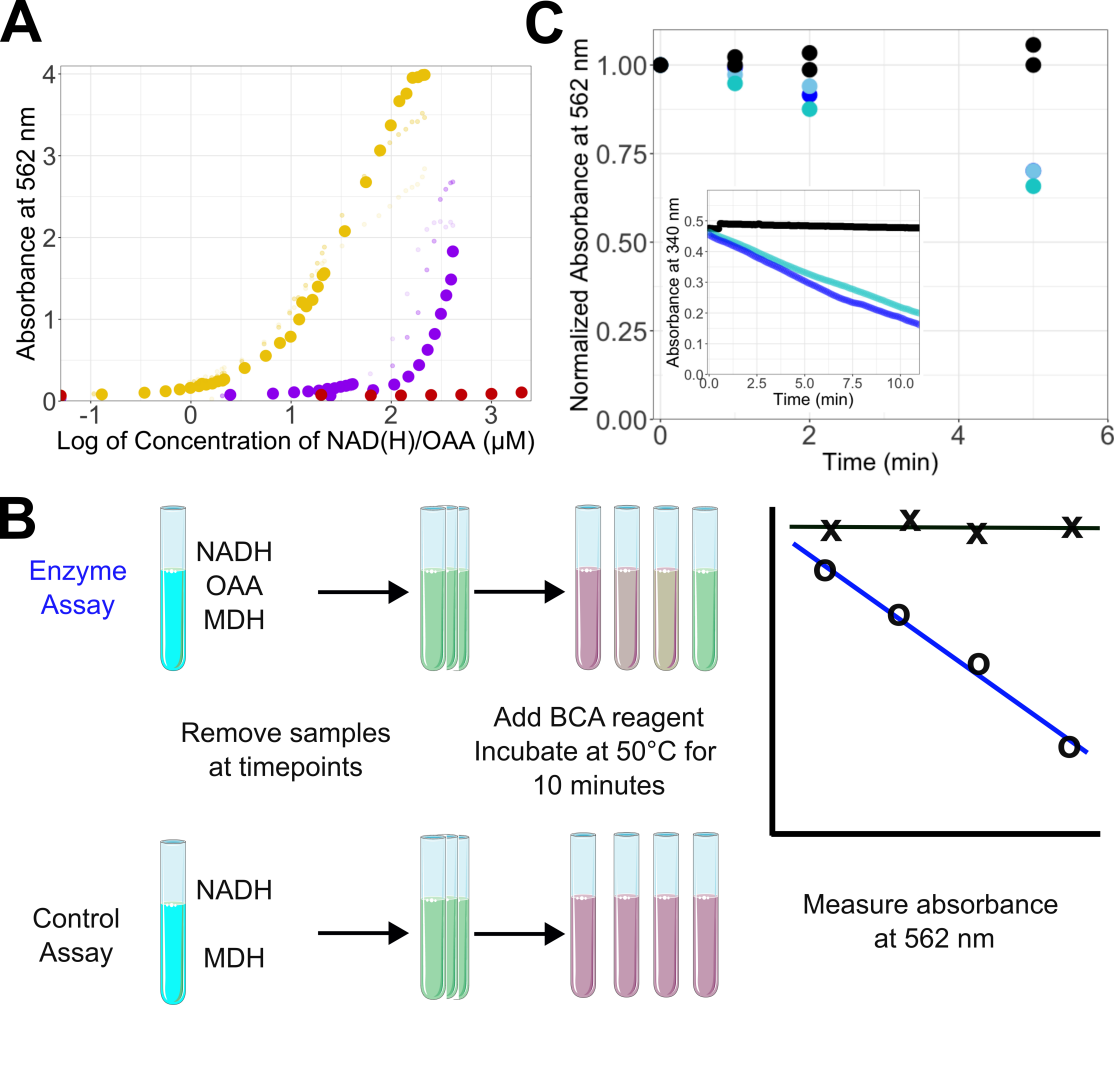
(A) Titration of NAD ^+^ (purple), NADH (gold), and oxaloacetate (red). The large dots are for assays performed with a 4:1 BCA reagent to sample ratio, while the smaller, lighter dots are for assays using a 1:1 and 2:1 reagent:sample ratio. (B) Overview of assaying MDH activity using the BCA assay (C) MDH assays using BCA reagent. The control assays containing no oxaloacetate are shown in black, while the replicate active enzyme assays are shown in shades of blue. The inset shows an absorbance assay at 340 nm under the same conditions as those used with the BCA reagent to detect activity. The data are representative of 5 separate trials performed by three individuals.

## Description

The bicinchoninic acid assay (BCA assay) is a common assay of protein concentration and reducing sugars (Garcia et al., 1993; Smith et al., 1985). The assay is based on the reduction of Cu(II) to Cu(I) by proteins or sugars, followed by the association of Cu(I) with bicinchoninic acid, which develops a visible purple color that can be measured at 562 nm (Huang et al., 2010; Smith et al., 1985; Wiechelman et al., 1988). While several small molecules are known to interfere with the reduction of copper, NAD ^+^ and NADH, which are associated with numerous redox reactions in cells, are not listed in the commercial kits (Brown et al., 1989). While we were attempting to assay the activity of beta-amylase in complex with malate dehydrogenase (MDH) using the BCA dye, we observed high BCA absorption in the presence of the MDH enzyme substrates. We then separately titrated NAD ^+^ and NADH into BCA dye, heated the mixture, and measured the absorbance at 562 nm ( Figure 1A ). We found that NADH exhibited a noticeably greater ability to reduce copper, resulting in a more pronounced color change than NAD ^+^ ( Figure 1A ). The absorbance of NAD ^+^ was dependent in part on the amount of BCA reagent added and could be suppressed by increasing the ratio of BCA dye to sample added. Under all conditions, NAD ^+^ produced less of a color change than NADH ( Figure 1A ). When we used 200 μL of BCA reagent with 50 μL of NAD(H) sample, we observed the largest separation in the concentration range where NAD(H) produced a color change. Under these conditions, the linear range where NADH produced a color change was 0 to 50 μM. At ~1 μM NADH, the difference between the background and sample absorbance was ~0.1, suggesting the potential for sub-micromolar detection of NADH using BCA dye.

Having demonstrated the differential effects of NAD+ and NADH in the BCA assay, we then set out to test whether we could utilize these differences to assay NAD(H)-dependent enzyme activity. Dehydrogenase enzymes are prevalent in biology and are of interest in both industrial and pharmaceutical industries (Baird et al., 2024; Fermaintt & Wacker, 2024; Martinez-Vaz et al., 2024; Parente et al., 2024; Reyes Romero et al., 2021; Springer et al., 2024). Thus, a simple and relatively inexpensive colorimetric enzyme assay for dehydrogenase enzymes could be helpful in instances when the traditional assay, which measures changes in absorbance by NAD(H) at 340 nm, is not feasible due to a lack of equipment or substances that absorb near 340 nm. During assay development and optimization, we first confirmed that the BCA reagent quenched the MDH reaction, finding no measurable activity above background when the BCA reagent was added before starting the assay. BCA working reagent from Fisher Scientific (catalog # PI23225) contains sodium hydroxide and has a pH of 10.5, which is outside the active pH range of many MDH enzymes; thus, the BCA working reagent should quench any enzyme activity (de Lorenzo et al., 2024). Because the enzyme can also react with the BCA working reagent, our control assays lacked oxaloacetate. Oxalocetate has no measurable reaction with copper or BCA dye relative to NADH at concentrations below 2 mM, which is the typical working maximum for most MDH assays, as MDH enzymes exhibit substrate inhibition by oxaloacetate (Bernstein & Everse, 1978; de Lorenzo et al., 2024; Shimozawa et al., 2022) ( Figure 1A ).

Having developed the conditions to ensure BCA absorbance at 562 nm was due to enzyme activity, we then tested whether we could measure MDH activity in the NADH and oxaloacetate to NAD ^+^ and malate direction. The general assay procedure is outlined in Figure 1B . The enzyme reaction and control reaction were assembled and incubated at 22°C. At appropriate time points, 50 μL of the enzyme or control reaction was removed and quenched in 200 μL of BCA on ice. After completion of the assay, all quenched reactions were incubated at 50°C for 10 minutes. After heating, 150 μL was transferred to the plate, and the absorbance was measured. As shown in Figure 1C, we observed a decrease in absorbance at 562 nm, consistent with the loss of NADH, in the presence of MDH and oxaloacetate, but not when oxaloacetate was lacking from the reaction mix. At the 5 minute mark, both types of assays had reduced absorbance by about 30% of the starting signal suggesting the BCA assay showed a comparable ability to detect enzyme activity as the traditional continuous 340 nm assay method.&nbsp;

Ultimately, these data show that the well-established BCA assay method can detect the presence of NADH with sensitivity comparable to the traditional spectroscopic assay method. Further, investigators should exercise caution when using BCA reagent in the presence of NAD(H)-containing solutions.

## Methods

BCA working reagent was prepared by mixing 50 mL of reagent A with 1 mL of reagent B. These reagents were a part of the kit from Fisher Scientific (catalog # PI23225). NAD ^+^ and NADH were prepared in distilled water, and their concentrations were determined using the extinction coefficient at 260 nm of 16,400 M ^-1^ cm ^-1^ . Oxaloacetate was prepared daily in 0.1 N HCl (Hatch & Heldt, 1985).

BCA assay data were collected using a Biotek Eon microplate reader at 562 nm in a 96-well plate. Typical well volumes were 150 μL. Stocks of NAD ^+^ or NADH in water or oxalacetate prepared in 0.1 N HCl were diluted in water to the assay concentration. Subsequently, 50-200 μL of each dilution was added to the BCA working reagent, and the samples were processed as follows for enzyme assays to develop the color change and collect data.

Enzyme assays were performed at pH 8 in 50 mM sodium phosphate and contained 200 μM oxaloacetate, 100 μM NADH, and 0.2 μM purified, recombinant human malate dehydrogenase 1. Assays were incubated in a water bath at 22 °C, and 50 μL was removed at the indicated time points in Figure 1C and put into 200 μL of BCA working reagent. Quenched reactions were left on ice until all the time points were collected. Quenched time points were then incubated at 50 °C for 15 minutes, after which 150 μL was transferred to a 96-well plate for data collection. Data were analyzed and plotted in R.
